# Functional Roles of Connexins and Gap Junctions in Osteo-Chondral Cellular Components

**DOI:** 10.3390/ijms24044156

**Published:** 2023-02-19

**Authors:** Agata Zappalà, Ivana Roberta Romano, Floriana D’Angeli, Giuseppe Musumeci, Debora Lo Furno, Rosario Giuffrida, Giuliana Mannino

**Affiliations:** 1 Department of Biomedical and Biotechnological Sciences, University of Catania, 95123 Catania, Italy; 2 Department of Human Sciences and Quality of Life Promotion, San Raffaele Roma Open University, 00166 Rome, Italy; 3 Department of Chemical, Biological, Pharmaceutical and Environmental Sciences, University of Messina, 98122 Messina, Italy

**Keywords:** Gap junctions, connexins, Cx43, osteocytes, osteoblasts, osteoclasts, chondrocytes, mesenchymal stem cells, bone, cartilage, homeostasis

## Abstract

Gap junctions (GJs) formed by connexins (Cxs) play an important role in the intercellular communication within most body tissues. In this paper, we focus on GJs and Cxs present in skeletal tissues. Cx43 is the most expressed connexin, participating in the formation of both GJs for intercellular communication and hemichannels (HCs) for communication with the external environment. Through GJs in long dendritic-like cytoplasmic processes, osteocytes embedded in deep lacunae are able to form a functional syncytium not only with neighboring osteocytes but also with bone cells located at the bone surface, despite the surrounding mineralized matrix. The functional syncytium allows a coordinated cell activity through the wide propagation of calcium waves, nutrients and anabolic and/or catabolic factors. Acting as mechanosensors, osteocytes are able to transduce mechanical stimuli into biological signals that spread through the syncytium to orchestrate bone remodeling. The fundamental role of Cxs and GJs is confirmed by a plethora of investigations that have highlighted how up- and downregulation of Cxs and GJs critically influence skeletal development and cartilage functions. A better knowledge of GJ and Cx mechanisms in physiological and pathological conditions might help in developing therapeutic approaches aimed at the treatment of human skeletal system disorders.

## 1. Introduction

Gap junctions (GJs) mediate intercellular communication through channels formed by connexins (Cxs). They allow a direct passage of electrical signals and small molecules up to 1.2 kDa (ions, second messengers, nutrients). In this way, they represent a tool for coordinating and regulating many aspects of cellular physiology, such as cell survival, metabolism and differentiation [1]. These intercellular channels typically consist of two opposed hemichannels (connexons), each formed by six Cxs. Cxs are polypeptides composed of four transmembrane domains, featuring an intracellular loop, two small extracellular loops, and intracellular amino and carboxyl terminal regions [2]. In homotypic channels, both connexons are made up of the same Cx subtype, whereas in heterotypic channels, each connexon contains different Cx subtypes [3]. In some instances, such as in myelinating glial cells, they can also connect cytoplasmic membranes of the same cell, forming autologous, or reflexive, GJs [4].

**Figure 1:**
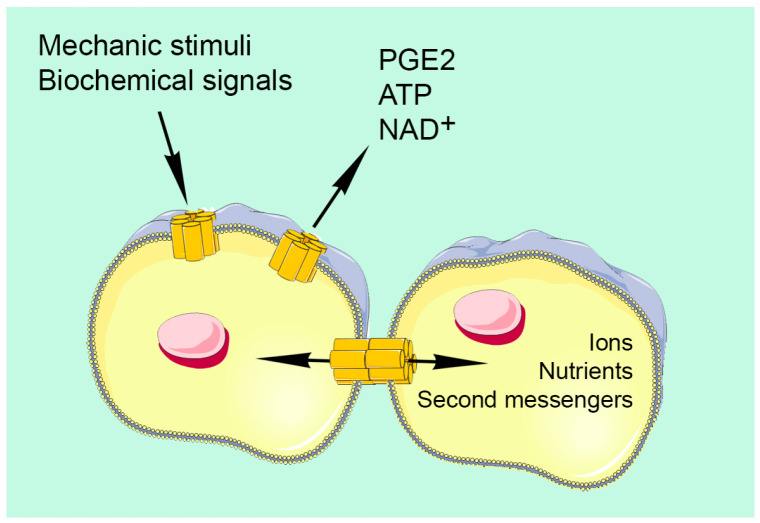
Schematic representation of gap junctions and hemichannels in bone cells.

Other than forming GJ channels between adjacent cells, undocked hemichannels provide a communication device between the intra- and extra-cellular environments (Figure 1). In addition, further communication channels, called pannexins, were discovered in vertebrates. Like connexins, they are able to form hemichannels [5], and three isoforms of pannexins have been identified in the mouse and human genomes [6,7].

Gap junctions consent intercellular transfer of small molecules, such as ions, nutrients, metabolites and second messengers (Ca^2+^ waves, IP3, cAMP). Hemichannels allow communication with the external environment; through hemichannels, cells may be influenced by extracellular cues (mechanic stimuli, biochemical signals), or release signaling molecules (PGE_2_, ATP, NAD^+^).

Cxs are present in cells of virtually all tissues (neurons, glial cells, cardiomyocytes, adipocytes, osteocytes, chondrocytes, etc.), with few exceptions (some neurons, red blood cells and platelets) [8,9,10]. Cxs are generally classified according to their molecular weight, largely ranging from 26 to 60 kDa; for example, Cx32 stands for a Cx protein of about 32 kDa. On the other hand, they can also be identified by their encoding genes, which include GJ, the homology groupings (A-E; according to the sequence identity and length of the cytoplasmic loop) and numerals based on the order of their discovery; for example, mouse Cx43 was the first Cx of the α-group to be discovered (Gja1), and mouse Cx32 was the first Cx of the β-group (Gjb1). To date, five connexin subfamilies have been identified (α, β, γ, δ and ε, or GJA, GJB, GJC, GJD and GJE), twenty-one Cx genes have been identified in the human genome, and twenty Cx genes have been identified in the mouse genome [2,11,12]. Depending on the Cx profile present in a particular cell type, GJs will determine different properties in terms of propagations of electrical signals and permeability to second messengers and metabolites, not only between interconnected cells but also between the intracellular milieu and the surrounding microenvironment [13]. This obviously plays a crucial role in cell physiological properties.

## 2. Bone Tissue

An extensive expression of GJs can be detected in the skeletal system, where they are fundamental to its development, homeostasis and plasticity. In bone tissue, GJs guarantee intercellular communication and coordination between the different types of cells (osteoblasts, osteoclasts and osteocytes) for bone growth, modeling and remodeling. Osteoblasts are responsible for new bone formation; osteoclasts are responsible for bone resorption; and osteocytes regulate the activation of the other two cell types [14]. Cx43 is the most diffusely present connexin in GJs of all types of bone cells and, because of its ubiquitous expression, it is the main candidate for the physiological network existing in bone tissue, especially between osteocytes and osteoblasts [11]. Indeed, other connexins (Cx26, Cx37, Cx40, Cx45 and Cx46) are also expressed, likely playing auxiliary roles (Table 1). It should be noted that Cx permeability may be different according to molecule size and charge. It was found that Cx43 permits the diffusion of relatively large molecules, with a preference for negatively charged particles. Cx40 and Cx26 significantly restrict the diffusion of anionic solutes, showing a preference toward molecules with a positive charge [15]. Cx37 is expressed in osteoclasts, osteoblasts and osteocytes and is required for osteoclast differentiation and fusion. Its absence prevents osteoclast maturation and leads to high bone mass and extracellular matrix (ECM) [16]. Cx40 seems important for the development of the forelimbs and sternum, but its expression in adult bone has not yet been demonstrated [17]. Cx45 expression is likely associated with matrix elaboration stages. However, its smaller pore size permits the passage of very small molecules with a molecular weight of less than 0.3 kDa; in fact, when heteromeric GJs include Cx45 and Cx43, the cellular permeability is reduced [18,19]. The functional role for Cx46 is still unknown; mainly expressed in osteoblastic cells, it is predominantly localized in the cytoplasmic trans Golgi network and cannot form GJs at the plasma membrane level [20,21]. It is possibly involved in osteoblast secretory pathways.

Besides osteoblasts, osteoclasts and osteocytes, other cell types such as chondrocytes and bone marrow stromal cells (BMSCs) play an active role in bone tissue physiology.

### 2.1. Osteoblasts

Osteoblasts are organized in a cellular monolayer on the bone surface and secrete osteoid components, which will eventually be mineralized. Osteoblasts are specialized in bone formation and remodeling [44]. In remodeling processes, osteoblasts control the activity of osteoclasts, which are responsible for bone resorption. In fact, the amount of bone resorbed by osteoclasts must be exactly replaced through osteoblast activity. The balance of these two processes is essential for bone homeostasis [59].

The presence of functional GJs has been demonstrated by in vitro studies both in murine and human osteoblasts [11]. Two GJ proteins have been identified in human osteoblasts: Cx43 and Cx45. Because of its lower permeability, Cx45 would be mainly responsible for electrical intercellular coupling, whereas Cx43 may be associated with a more elevated degree of metabolic exchanges [34]. Cx43 is diffusely expressed and its expression increases during osteoblastic differentiation [23]. Data available in the literature clearly show Cx43 involvement in correct skeletal development and acquisition of bone mass. In fact, it plays a key role in the expression of osteoblast-specific promoters such as osteocalcin and bone sialoprotein, critical genes for bone matrix formation and calcification [24]. Through experiments in mice, Lecanda et al. [25] showed that the mineralization potential of osteoblasts in Cx43-null embryos is strongly impaired. In these animals, although axial and appendicular bone segments were essentially normal at birth, both endochondral and intramembranous ossification of the cranial vault were delayed, associated with a retarded ossification of the vertebrae, clavicle, ribs and limbs; in these cases, the impaired intercellular diffusion of calcein further supports the importance of GJs in maintaining osteoblast function in skeletal development. Supporting evidence was provided by experiments on miR-206, which negatively affects Cx43 expression. In a study by Inose et al. [26], it was shown that osteoblast differentiation is negatively influenced by miR-206, whose expression declines along with osteoblast differentiation. As expected, transgenic mice expressing miR-206 in osteoblasts developed a low bone mass phenotype due to impaired osteoblast differentiation, which was enhanced by knocking down miR-206 expression. These observations were corroborated by recent investigations [27], showing a significant reduction of osteogenesis after knocking down circAKT3, a circular RNA that interferes with the expression of miR-206. It was concluded that circAKT3 can enhance osteogenesis by eliminating the suppressive effect of miR-206 on Cx43. It was also reported that the treatment of osteoblastic cells with 18-α-glycyrrhetinic acid and oleamide, two nontoxic reversible GJ inhibitors, not only hampered their differentiation into mature osteoblasts, but rather induced a trans-differentiation towards adipocyte-like cells [23]. This is not, however, surprising, since adipocytes and osteoblasts are assumed to differentiate from common stromal progenitor cells, and GJ expression is downregulated during adipogenesis [60,61].

Numerous anabolic factors, such as bone morphogenetic protein 2 (BMP2), Prostaglandin E_2_ (PGE_2_) and parathyroid hormone (PTH), upregulate Cx43 protein and GJ communication [35]. PTH-activated signaling induces a feed-forward mechanism that enhances Cx43 expression. This, in turn, amplifies the ability of osteoblasts to respond to PTH, facilitating bone growth associated with the diffusion of cAMP from osteoblasts to osteocytes [36]. This is important not only in normal conditions but also during bone healing after fracture [28]. When Cx43 expression was disrupted using antisense RNA, the response to PTH was weakened, and a significantly reduced PTH-induced matrix mineralization occurred in mature osteoblasts [13].

Besides GJs, osteoblasts express Cx43 functional hemichannels, as indicated by the cellular uptake of Lucifer yellow dye and inositol triphosphate [34]. Cx43 GJs and hemichannels are essential for determining the normal responsiveness of osteoblast to biochemical and physical stimulation.

### 2.2. Osteocytes

Osteocytes and osteoblasts are the most numerous cell types of bone tissue (up to 95%). Osteocytes form bone tissue by two mechanisms: endochondral and intramembranous ossification. In endochondral ossification, in areas destined for bone formation, precursor cells condense and acquire the shape of the bone segment, acting as a cartilaginous template [62,63]. This mechanism predominantly occurs for most skeleton elements, such as the skeletal axis, limbs and the basal/caudal part of the skull. The other skull parts, such as the cranial vault and the maxillomandibular bones, are formed by intramembranous ossification. In this case, bone tissue originates from the condensation of mesenchymal precursors, which directly differentiate into bone cells, without a transitional cartilaginous template. Unlike osteoblasts and osteoclasts that last only days or weeks, osteocytes can survive several years [64].

**Figure 2:**
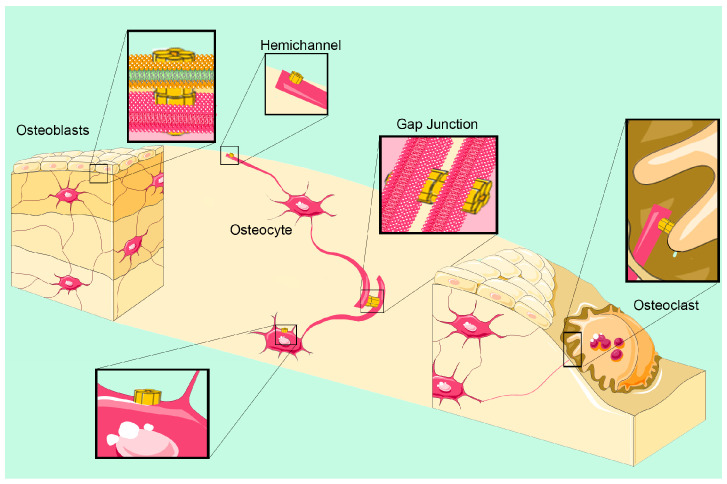
Schematic representation of Cx43 gap junctions and hemichannels in bone cells. Through gap junctions located in their long dendritic processes, osteocytes embedded in the deep lacunae are able to make connections with each other and with other bone cells such as osteoblasts and osteoclasts, in spite of the surrounding mineralized matrix. The resulting “functional syncytium” allows diffuse intercellular propagation of metabolites, second messengers and Ca^++^ waves. Through hemichannels, osteocytes may sense a variety of extracellular cues (mechanic stimuli, biochemical signals (hormones, growth factors, cytokines)), or release signaling molecules (PGE_2_, ATP, NAD^+^) into the external environment.

The massive expression of Cx43 in osteocytes allows an extensive GJ formation not only with each other, but also between osteocytes and osteoblasts (Figure 2). To accomplish their role, it is fundamental that osteocytes, although being embedded deep in lacunae of bone tissue, are able to sense and/or diffuse biochemical signals also at considerable distances, in spite of the surrounding mineralized matrix. This is made possible by the presence of long dendritic-like processes that, through canaliculi in the bone tissue, enable physical interconnections between neighboring osteocytes as well as bone cells even located at the bone surface (osteoblasts and osteoclasts). As a result, through Cx43 GJs, a widespread “functional syncytium” is generated, which allows “prisoner” osteocytes to act as “orchestrators” of bone processes [29].

Among bone cells, it is generally recognized that osteocytes are the principal cells acting as mechano-sensors [30]. Osteocytes and their processes are surrounded by a fluid-filled environment that extends through the lacuna–canaliculi network. This interstitial fluid is a major stress-related factor that transmits mechanical stimulation to other GJ-interconnected bone cells. Mechanical loading produces a movement of the interstitial fluid that is sensed by the osteocytes through integrins, cilia, calcium channels, and G-protein coupled receptors, which act as mechano-sensors. Together with the opening of Cx43 hemichannels [31], mechanical stimuli are transduced in biochemical signals that are transmitted through the lacuna–canaliculi network not only to interconnected osteocytes, but also to osteoblasts, osteoclasts and bone lining cells. In particular, it has been reported that during mechanical stimulation, PGE_2_ is produced in a GJ-dependent manner by osteocyte-like cells and, by a feed-forward mechanism, an increased PGE_2_ production further increases Cx43 expression. GJs located at the extremities of the long dendritic processes are crucial devices for this communication network.

### 2.3. Osteoclasts

When compared to osteoblasts and osteocytes, little evidence exists for intercellular osteoclast communication. However, data showing Cx43 expression in GJ communication have also been reported for these cells [37,38]. Osteoclasts are multinucleated cells originating from the fusion of monocyte-like precursor cells, and Cx43 appears involved in the fusion process. Using the pit formation assay, an impaired precursor fusion was observed following treatment with heptanol, a known GJ inhibitor: the number of osteoclast-like cells was significantly reduced, whereas the number of unfused, mononuclear precursor cells increased. Moreover, the fewer multinucleated osteoclasts obtained by this treatment showed a reduced activity, as the total resorbed area and the number of resorption pits also decreased in the cultures tested. Similar results were obtained using a synthetic connexin-mimetic peptide, Gap 27, a more specific GJ inhibitor [65]: Gap 27 treatment caused a marked reduction of both mononuclear and multinucleated rat osteoclasts, cultured on bovine bone slices. In addition, a decreased cell survival was reported for osteoblast-like cells, not related to possible Gap 27 toxic effects, since the other cells in the culture were largely unaffected. The above observations were also confirmed by Ransjö et al. [39] using the GJ inhibitors 18-α-glycyrrhetinic acid and oleamide. In this work, reabsorption pits were reduced, associated with a reduced response of osteoclast activity to vitamin D3 and PTH. Moreover, in bone marrow cultures, osteoclast differentiation induced by PTH and vitamin D3 was reduced following GJ inhibition with carbenoxolone [40], and the same inhibitor significantly prevented osteoclastogenesis stimulated by the receptor activator of NF-kappaB ligand (RANKL).

### 2.4. Connexin-Mediated Bone Tissue Plasticity

A body of evidence demonstrates that GJs are crucial devices underlying bone growth, modeling and remodeling. During development, it has been reported that Cx43 expression is 80-fold higher in neonatal mice as compared to adult mouse bone marrow [66], and this is likely associated with the extensive communication between bone marrow stromal and stem cells. The critical role of Cx43 in limb growth was demonstrated by investigations in chick embryo development, where the inhibition of Cx43 expression led to truncation and malformation of limbs [67,68]. When Cx43 antisense oligonucleotides were applied in cells of early chick facial primordia, mandibular bone formation in the embryonic chick was significantly reduced, along with substantial facial defects [69].

Bone tissue plasticity is particularly evident for the mechano-transduction ability of osteocytes that enable the skeleton to respond to mechanical stresses, adapting the tissue microarchitecture to the changing demands of mechanical loads. Mechanical stimuli activate stretch-gated receptors, leading to the opening of Cx43 hemichannels in osteocytes. Although molecular mechanisms triggering the intracellular signaling cascade are not fully elucidated, available data suggest that integrins may initiate the cellular response acting as mechanosensitive molecules [70,71], and some soluble factors like ATP, NO and PGE_2_ are released by osteocytes in response to mechanical stimuli [72,73]. This was shown in MLO-Y4 osteocyte-like cells, where PGE_2_ release would occur by opening Cx43 hemichannels, following a physical interaction between Cx43 hemichannels and β1 integrins. In fact, inhibition of β1 integrin prevents PGE_2_ release after mechanical stimulation [70]. It is worth noting that mechano-induced NO release promotes bone formation and inhibits resorption. Mechanical stimulation also triggers an intracellular calcium wave that propagates through the osteocyte syncytial network and is transmitted to the other connected cells, such as osteoblasts [74,75,76]. Many studies have highlighted the relevance of Cx43 on these mechano-induced responses, which would balance osteo-anabolic or osteo-catabolic responses that, under different conditions, can be differently orchestrated in the different bone locations, such as the periosteal or the endosteal regions [32,33]. For example, even in homeostatic conditions, it has been proposed that Cx43 can modulate mechano-induced bone modeling, limiting both endocortical bone resorption and periosteal bone formation.

Another important Cx43-mediated effect relates to osteocyte apoptosis. This is of great interest since an increased osteocyte death is associated with various catabolic bone syndromes, where an increased osteocyte apoptosis leads to increased bone resorption and reduced bone mass, also impairing communication among bone cells [77]. Experimental data have highlighted the anti-apoptotic role of Cx43 and downstream signaling in osteoblasts and osteocytes [41]. These authors showed that Cx43 is required for the efficacy of the bone anabolic therapeutic drug alendronate, which prevents etoposide- and dexamethasone-induced apoptosis on osteoblasts. Dye uptake tests report that alendronate is able to open Cx43 hemichannels of MLO-Y4 osteocyte-like cells, also demonstrating that the Src-ERK signaling cascade mediates this antiapoptotic effect. Src is a proto-oncogenic protein involved in cell growth and differentiation regulation. The role of Cx43 hemichannels as receptors for bisphosphonates was demonstrated in cells cultured at low density or in suspension, where GJ intercellular communication is minimized. In synthesis, they propose that bisphosphonates induce the opening of Cx43 hemichannels, resulting in the subsequent activation of Src. This would in turn stimulate the mitogen-activated protein kinase cascade, eventually leading to ERK phosphorylation and cell survival. The effects would be amplified in normal conditions, where GJ intercellular communication occurs and the intracellular signals triggered by the “primary cellular response” can be transmitted from one cell to another [13].

## 3. Cartilage

In the skeletal system, cartilage is responsible for two essential functions: as a scaffold for endochondral ossification and as articular cartilage for frictionless joint movements [46]. In both cases, GJ-mediated intercellular connections between chondrocytes and the environment or surrounding cells play a crucial role, allowing metabolic exchanges of nutrients and signaling molecules. Forming three-dimensional networks, they are fundamental to maintaining cartilage homeostasis, providing a synchronized regulation of chondrocyte physiologic activity [47].

Human chondrocytes express Cx43, Cx45, Cx32 and Cx46 [1,78]. However, as for the bone tissue, Cx43 is the most abundant, both in GJs and in undocked hemichannels. Hemichannels also play important roles, acting as receptors from the external environment (i.e., mechanical stimulation, growth factors and cytokines), and releasing paracrine signals such as ATP or NAD^+^. Immunohistochemical investigations showed Cx43 distribution in hyaline cartilage and in the perichondrium of mouse and rat knee joints, where coupled chondrocytes were demonstrated by the Lucifer yellow transfer test [79]. The authors conclude that Cx43 likely plays a major role during development, since a lower expression of this connexin was found in the hyaline cartilage of mature rats. Other Cxs are, however, needed, since Cx32 is required for a normal limb bud development [22].

During endochondral ossification, cartilage templates guide the formation of most bones of the skeleton, especially long bones [5]. The process begins with mesenchymal cell condensation, followed by proliferation and differentiation. Chondrocytes located in the growth plates at both sides of the bone are organized in a columnar pattern at different steps of differentiation. In the most distal part, resting chondrocytes are found in the hyaline cartilage, whereas underlying proliferative chondrocytes continuously divide by mitosis. Facing the epiphysis, proliferative chondrocytes push older cells toward the diaphysis, where they progressively differentiate into pre-hypertrophic, hypertrophic and mature chondrocytes. Mature chondrocytes eventually degenerate as ECM becomes calcified by osteoblast activity. In this way, bone length is extended at both ends, at least until growth plate fusion. Immunohistochemical investigations revealed Cx43 and high levels of Cx43 mRNA expression in chondrocytes involved in endochondral ossification [79,80]. The importance of Cx43 for chondrocyte differentiation was underlined by results obtained in experiments using in vitro micro mass cultures of chondrocytes from the chick limb bud [45]. It was demonstrated that GJ inhibition with 18-α-glycyrrhetinic acid reduced the production of proteoglycans and type II collagen. Moreover, GJ inhibition impaired BMP2-induced chondrocyte anabolic effects and differentiation. In another study, during chondrogenic differentiation of cultured chick leg bud mesenchymal cells, TGF-b3 treatment downregulated Cx43 mRNA expression and induced apoptotic cell death via downregulation of integrin b4, activation of ERK and suppression of PKC-a activation [42]. Some controversial results were obtained in vivo from Cx43-null mice, indicating that Cx43 might not necessarily be required for bone growth during embryogenesis [25]. As mentioned previously, although both intramembranous and endochondral ossification of the cranial vault were delayed, the axial and appendicular bone segments of Cx43-null animals were basically normal at birth if compared with wild-type animals. A possible explanation for the discrepancy between these in vivo and in vitro studies is that other Cxs can compensate Cx43 inhibition. For example, it has been suggested that Cx40 also plays a role in the regular development of sternum and forelimb bones, since its deficiency is responsible for many skeletal malformations [17]. In particular, these authors demonstrated that, at least in part, T-box transcription factor 5 exerts its regulatory role in bone growth and maturation by controlling, through Cx40, the expression of Sox9, a transcription factor that is essential for chondrogenesis and bone growth. 

Articular cartilage is a highly specialized avascular alymphatic connective tissue that, thanks to its special mechanical properties, provides a smooth surface that allows the painless movement of joints [47]. Articular chondrocytes are responsible for producing and maintaining the dense ECM at the epiphysis of long bones. It acts as a biomechanical shock absorber because of its content of massive amounts of collagen, proteoglycans and water, which alleviate the load between bones. Within the dense ECM, articular chondrocytes are embedded in small cavities called “lacunae” and are connected with each other by at least two long cytoplasmic projections that reach distant cells located in different lacunae. In this way, a functional interconnection between different chondrocytes may occur through functional GJs formed by Cx43, as was demonstrated in cultures of articular chondrocytes by dye transfer tests [46,79,81,82]. In this respect, it should be noted that the development of chondrocytic cell lines such as the T/C-28a2 cell line has permitted investigations on cartilage properties, exploring both physiological mechanisms and physiopathological characteristics of cartilage diseases.

In articular chondrocytes, GJ communication is particularly important for coordinating both the metabolic activity and the sensitivity to extracellular stimuli such as mechanical loads on the joint. Cyclic compression opens chondrocyte hemichannels, triggering ATP release into the extracellular milieu activating purinergic receptors. ATP release in these conditions was blocked by the hemichannel inhibitor flufenamic acid [83,84,85]. When single articular chondrocytes are stimulated by a mechanical perturbation, an intercellular Ca^2+^ wave propagation occurs between adjacent chondrocytes, also involving synovial fibroblasts [48,86,87]. The role of functional GJs in this process was assessed by the reduction of intercellular Ca^2+^ spreading following treatment with a GJ inhibitor (18-aglycyrrhetinic acid). Although the events occurring in the transduction of mechanical to biochemical signals are not fully understood, it has been proposed that an initial increase of intracellular calcium concentration in chondrocytes may result from deformation-activated mechanosensitive ion channels [49]. The consequent brief depolarization would stimulate Ca^2+^-activated K+ channels, leading, in turn, to hyperpolarization. This would trigger a positive-feedback loop, by which a further influx of calcium occurs. Alternatively, an increased cytosolic calcium concentration would result from an increased release from intracellular stores in the endoplasmic reticulum. Other results show that mechanical stimulation activates phospholipase C, leading to an increase of intracellular inositol 1,4,5-trisphosphate. This second messenger, passing through GJs, stimulates intracellular Ca^2+^ release in adjacent chondrocytes, thus amplifying the response [48].

Cx43-mediated intercellular communication between cartilage, subchondral bone and synovial tissue suggests a molecular crosstalk between the various tissues of the joints [1]. Synovial fibroblasts are mesenchymal-derived cells, which form a thin layer of synovial tissue contiguous to the fibrous capsule of the joint. Synovial tissue produces synovium, a fluid that lubricates the joints and supplies nutrients to articular chondrocytes. Alterations of this communication network are assumed to be responsible for the etiology of osteoarthritis, which is characterized by ECM degradation and cartilage destruction. In fact, osteoarthritis would not simply result from wear-and-tear processes, but rather from altered biochemical and molecular crosstalk between the tissues involved, leading to pathological changes that induce the progressive destruction of articular cartilage. [43,87,88]. In osteoarthritis, synovial fibroblasts would increase the release of inflammatory cytokine such as interleukin-1 (IL-1), which is considered one of the most prevalent catabolic factors leading to cartilage destruction [89]. In fact, IL-1 upregulates Cx43 expression in cultured chondrocytes, and pathological increases of Cx43 expression are observed in both synovial fibroblasts and articular chondrocytes in osteoarthritis [50,51]. Electron microscopic observations confirmed an increased size and number of Cx43 GJs between synovial lining cells of osteoarthritic patients compared with healthy subjects [90]. Moreover, in vitro experiments showed that blocking GJs with 18-α-glycyrrhetinic acid or octanol decreases IL-1-stimulated synovial fibroblast production of metalloproteinases that degrade cartilage ECM [91]. It can be speculated that GJ communication would amplify catabolic signals triggered by mechanical perturbation on synovial fibroblasts. The subsequent production of IL-1, in turn, upregulates Cx43 expression in both synovial fibroblasts and articular chondrocytes, further increasing the production of catabolic factors such as metalloproteinases, IL-1 and other cytokines [13]. GJ-mediated intercellular calcium signaling between articular chondrocytes and synovial cells would indeed be involved, since this process can be prevented by intracellular Ca^2+^ chelation.

Immunohistochemical experiments in cartilage from osteoarthritis patients showed significant increases of Cx43 levels [78], from the superficial zone down to nearly 1 mm of tissue, the particularly damaged regions. According to Varela-Eirin et al., [52], Cx43 acts as a positive regulator that reverts chondrocytes to a less differentiated state, possibly by upregulating the activity of the basic helix–loop–helix transcription factor Twist-1. However, overactive Cx43 would maintain the immature phenotype by increasing nuclear translocation of Twist-1 and would increase tissue remodeling by increasing metalloproteinases. Moreover, increased production of proinflammatory agents and IL-1 would contribute to cellular senescence. In this context, it has been shown that carbenoxolone-induced downregulation of either Cx43 or Cx43-mediated intercellular communication may trigger dedifferentiation of osteoarthritic chondrocytes into a more differentiated state, associated with decreased synthesis of MMPs and proinflammatory factors.

## 4. Bone Marrow Mesenchymal Stem Cells (BMSC)

Bone tissue cells interact with various types of stem cells that are present in bone marrow niches [92]. Hematopoietic stem cells, so called for their ability to form all blood cells, were the first population of adult stem cells to be identified [93]. A second population with different characteristics was discovered a few years later [94]. Originally called “bone marrow stromal cells”, they consist of a mixed population that, besides supporting hematopoiesis [95], also feature self-renewal capability, high proliferative potential and the ability to differentiate into mesodermal elements, such as chondrocytes, osteoblasts and adipocytes [96]. For this reason, they are considered mesenchymal stem cells.

Data available suggest that these bone marrow cells are connected through GJs, as indicated by the transfer of Lucifer yellow from single cells to most other, electrotonically coupled adjacent stromal cells [97]. In particular, it was also shown that Cx43, rather than other connexins, was present in these GJs, and treatment with IL-1 resulted in a reversible decrease of this transfer ability. Together with osteocytes and bone lining cells, BMSCs form a sort of “bone basic cellular system”, which allows a continuous cytoplasmic network extending from osteocytes to endothelial cells. This system would able to sense mechanical and biochemical stimuli, and then trigger processes of bone formation and/or resorption [29]. GJ mediated intercellular communication is involved in multiple BMSC activities. For example, it was shown that Cx43 and Cx45 GJs mediate the secretion of CXCL12, an essential chemokine for hematopoietic stem cell function [98]. Other studies suggest Cx43-mediated effects on the balance between proliferation and differentiation of hematopoietic precursors [99]. BMSCs can mainly differentiate into osteoblasts and adipocytes [54,55,100] following specific stimulation. For example, PTH administration stimulates osteoblastic differentiation, thus increasing the osteoblast population. Recent results show that Cx43 and GJs play a role during BMSC progressive commitment and differentiation toward osteoblast progenitors, immature osteoblasts and mature osteoblasts. The authors claim that Cx43-mediated propagations of intracellular Ca^2+^ oscillations are crucial in the regulation of these differentiation steps [56]. Overexpression of Cx43 enhances osteogenic differentiation of these cells and their transplantation in nude mice resulted in an increased volume fraction and spatial uniformity of bone in vivo [57]. The increased GJ expression also enhanced osteoinductive effects of BMP-7, suggesting a synergism between GJs and this soluble factor. Moreover, GJs were also indicated as the probable device for enhancing the BMSC osteogenic potential promoted by Panax notoginseng, the Chinese medicinal herb that has long been used to treat bone fractures [58].

The ability of BMSCs to differentiate into bone cells has prompted their use in transplantation studies in regenerative medicine-based applications [101]. In fact, an increasing number of BMSC-based therapies are being carried out for the treatment and repair of musculoskeletal tissue diseases, as evidenced by numerous human clinical studies addressing various bone regeneration applications (i.e., repair of long bones and vertebrae fractures, repair of craniofacial bone, treatment of bone-related diseases such as osteogenesis imperfecta). Attention in this field has been recently focused on the osteogenic differentiation ability of adipose-derived mesenchymal stem cells (ASCs) [102,103]. Indeed, ASCs offer numerous advantages and can be easily harvested for autologous transplantation [104]. Moreover, by appropriate differentiation strategies, they can give rise not only to mesodermal elements [105,106,107], but also to neural-like cells [108,109]. Because of these properties, ASCs, as well as BMSCs, might be considered a valuable tool in regenerative medicine-based therapeutic applications [110,111,112]. The expression of various Cxs has been detected in naïve ASCs [113], also showing that Cx expression patterns may vary according to their differentiation pathway towards different cell types (neurons, glial cells or adipocytes). It is important to underline that, besides Cx32, Cx36 and Cx47, Cx43 was found to be the most abundant Cx in basal conditions, and this is particularly interesting since, as has been frequently stressed, Cx43 is the most expressed connexin in skeletal system tissues.

## 5. Conclusions

GJ intercellular communication is fundamental for skeletal system physiology. It is primarily involved in coordinating bone tissue responses to external stimuli, such as mechanical stimulation or biochemical signals such as growth factors and hormones. In addition, GJs mediate the effects of local molecules (cytokines, growth factors and prostaglandins) in the balance between bone resorption and formation. Other than paracrine activities, GJ-mediated intercellular communication represents a further valuable mechanism for coordinating tissue physiology. In this regard, Cx hemichannels can be considered as intermediate devices sharing some properties of these two mechanisms: they may act as receptors to external stimuli and allow trans-membrane transfer of biochemical signals into the surrounding environment. GJ-mediated communication is essential to coordinate the activity not only between bone tissue cells (osteoblasts, osteocytes and osteoclasts), but also between cells of other tissues involved (cartilage, synovial tissue). It is important to emphasize that increasing knowledge of GJ mechanisms and connexin participation is fundamental not only to better comprehend important aspects of cell biology and human physiology, but also to help develop therapeutic approaches for human pathologies. For example, enhancing Cx43 expression may improve bone formation during fracture healing [28], and targeting chondrocyte plasticity via Cx43 modulation would help cartilage regeneration in osteoarthritis [53].

## Figures and Tables

**Table 1:** Connexins (Cxs) mainly expressed in osteo-chondral cellular components.

Connexin	Human Gene	Mouse Gene	Cells	Functions	References
Cx26	GJB2	Gjb2	Bone cells	It restricts the diffusion of anionic solutes and facilitates the transfer of positively charged molecules	[15]
Cx32	GJB1	Gjb1	Chondrocytes, mesenchyme cells	Normal limb bud development	[22]
Cx37	GJA4	Gja4	Bone cells	Osteoclast differentiation	[16]
Cx40	GJA5	Gja5	Bone cells, chondrocytes	It participates in the development of the sternum and forelimb bones, although its expression in adults has not yet been demonstrated	[17]
Cx43	GJA1	Gja1	Bone cells, chondrocytes	It is permeable to relatively large molecules, with a weak preference for negatively charged particles	[15]
				Osteoblast differentiation and extracellular matrix mineralization	[23,24,25,26,27,28]
				Interconnections between osteocytes and between osteocytes with osteoblasts and osteoclasts, arranging a “functional syncytium” within bone tissue	[11,29]
				Transduction of mechanical into biochemical signals that are propagated through bone tissue by the syncytial network	[30,31,32,33]
				Cell response to biochemical signals from the external medium	[28,34,35,36]
				Osteoclastogenesis. Osteoclast reabsorption activity	[16,37,38,39,40]
				Anti-apoptotic effects	[13,14,41,42]
				Interactions between articular cartilage and subchondral bone	[43]
				Chondrocyte differentiation	[42,44,45]
				Arrangement of the articular chondrocyte network	[1,46,47]
				Intercellular propagation of Ca2+ waves following mechanical stimulation of articular chondrocytes	[48,49]
				Implicated in the etiology of osteoarthritis	[13,50,51,52,53]
			Bone marrow stromal cells	Osteogenic differentiation	[54,55,56,57,58]
Cx45	GJC1	Gjc1	Osteoblasts, chondrocytes	Because of its small pore, it is mainly responsible for intercellular electrical coupling	[18,19]
Cx46	GJA3	Gja3	Osteoblasts, chondrocytes	Localized in the cytoplasmic trans Golgi network, it cannot participate in channels at the plasma membrane level. It is possibly involved in osteoblast secretory pathways	[20,21]

## Data Availability

No further data are available.

## Abbreviations

AKT	Akt serine/threonine kinase
BMP	Bone morphogenetic protein
BMSCs	Bone marrow stromal cells (103)
Cx	Connexin
CXCL12	C-X-C motif chemokine ligand 12
ECM	Extracellular matrix
ERK	Extracellular signal-regulated kinase
GJs	Gap junctions
HC	Hemichannel
IL-1	Interleukin-1
IP3	Inositol trisphosphate
PGE2	Prostaglandin E2
PKC	Protein kinase C
PTH	Parathyroid hormone
RANKL	Receptor activator of nuclear factor kappaΒ ligand
TGF	Transforming growth factor
